# Thrombopoiesis meets tumorigenesis: unlocking the diagnostic and prognostic potential of platelet indices in breast cancer– a narrative review

**DOI:** 10.1097/MS9.0000000000004714

**Published:** 2026-01-21

**Authors:** Emmanuel Ifeanyi Obeagu

**Affiliations:** a Department of Biomedical and Laboratory Science, Africa University, Mutare, Zimbabwe; b Department of Molecular Medicine and Haematology, Faculty of Health Sciences, University of the Witwatersrand, Johannesburg, South Africa

**Keywords:** breast cancer, platelet indices, prognostic biomarkers, thrombopoiesis, tumor microenvironment

## Abstract

Breast cancer, as the most frequently diagnosed malignancy in women, continues to challenge clinicians due to its heterogeneity and variable clinical outcomes. As traditional diagnostic and prognostic markers often fall short in fully capturing disease dynamics, there is growing interest in the role of hematological biomarkers, particularly platelet indices, as accessible and cost-effective tools in cancer management. Recent evidence suggests that thrombopoiesis is not merely a bystander process but actively contributes to tumorigenesis through complex interactions with cancer cells and the tumor microenvironment. Platelets influence breast cancer progression by promoting angiogenesis, shielding circulating tumor cells from immune clearance, and facilitating metastasis. These tumor-promoting functions are reflected in measurable changes in platelet indices, such as platelet count, mean platelet volume, platelet distribution width, and plateletcrit. Abnormal values of these indices have been associated with advanced tumor stage, lymph node involvement, and reduced survival, highlighting their potential as biomarkers for disease monitoring and prognostication. Nevertheless, with further validation and integration into multimodal diagnostic frameworks, platelet-based biomarkers may enhance precision in breast cancer risk stratification and treatment planning. This review underscores the need for continued investigation into the interplay between thrombopoiesis and tumorigenesis and the translational potential of platelet indices in breast cancer care.

## Introduction

Breast cancer is the most frequently diagnosed cancer and a leading cause of cancer-related mortality among women worldwide^[1,2]^. Despite significant advances in early detection and therapeutic strategies, the heterogeneity of breast cancer continues to pose challenges for prognosis and individualized treatment. Traditional prognostic tools – such as tumor grade, stage, receptor status, and molecular profiling – provide valuable insights but may not fully capture the dynamic biological processes underpinning disease progression. Therefore, the search for novel, cost-effective, and minimally invasive biomarkers remains a priority in optimizing breast cancer management^[3–6]^. Among the emerging candidates are platelet indices, routinely available parameters derived from complete blood count tests, including platelet count (PLT), mean platelet volume (MPV), platelet distribution width (PDW), and plateletcrit (PCT). Historically relegated to the assessment of bleeding and thrombotic disorders, these indices are now gaining attention in oncology due to their potential to reflect systemic inflammation, tumor-induced hematopoietic alterations, and platelet activation – all of which are intricately involved in cancer biology^[7–10]^. The relationship between breast cancer and thrombosis has long been recognized, with platelet hyperactivity and paraneoplastic thrombocytosis reported in various malignancies, including breast cancer. Tumors secrete inflammatory cytokines such as interleukin-6 (IL-6), which stimulate thrombopoiesis through hepatic thrombopoietin production and bone marrow megakaryopoiesis. As a result, elevated platelet counts may reflect a tumor-driven inflammatory state and correlate with more aggressive disease phenotypes^[11–14]^.

HIGHLIGHTS

- Explores the interplay between thrombopoiesis and breast cancer progression.
- Highlights how platelet indices reflect tumor burden and metastatic potential.
- Emphasizes mean platelet volume as a key biomarker.
- Discusses platelet–tumor cell interactions in immune evasion and angiogenesis.
- Suggests clinical utility of platelet indices in prognosis and treatment monitoring.

Beyond their role in hemostasis, platelets are increasingly understood as active modulators of tumor progression. They secrete a wide array of bioactive molecules – such as vascular endothelial growth factor (VEGF), platelet-derived growth factor (PDGF), and transforming growth factor-beta (TGF-β) – that promote angiogenesis, tumor cell proliferation, and immune evasion. Moreover, platelets facilitate epithelial-to-mesenchymal transition (EMT) and protect circulating tumor cells (CTCs) from immune-mediated destruction, thereby aiding metastasis^[15,16]^. These functional attributes of platelets suggest that changes in their quantity and quality, as reflected by platelet indices, may serve as surrogates for underlying oncogenic processes. Studies have demonstrated that abnormal MPV and PDW values are associated with lymphovascular invasion, higher tumor grade, and poorer survival outcomes in breast cancer patients. Similarly, elevated PLT and PCT levels have been linked with advanced-stage disease and resistance to chemotherapy, underscoring their potential prognostic significance^[17,18]^.

### Aim

This narrative review aims to explore the intricate relationship between thrombopoiesis and tumorigenesis, with a particular focus on the diagnostic and prognostic relevance of platelet indices in breast cancer.

## Methods

This narrative review was conducted to synthesize current evidence on the role of platelet indices in breast cancer, focusing on their diagnostic, prognostic, and therapeutic implications. A systematic approach was employed to ensure comprehensive coverage and transparency.

## Literature search strategy

A structured literature search was performed using the electronic databases PubMed, Scopus, and Web of Science. The search included studies published between January 2000 and June 2025. Keywords and Medical Subject Headings (MeSH) terms included: “platelet indices,” “platelet count,” “mean platelet volume,” “platelet distribution width,” “plateletcrit,” “tumor-educated platelets,” “tumor RNA,” “breast cancer,” and “prognosis.” Boolean operators (AND, OR) were used to refine the search.

## Inclusion and exclusion criteria

Studies were included if they:

1. Investigated platelet indices in breast cancer patients;
2. Reported diagnostic, prognostic, or therapeutic outcomes;
3. Were published in English;
4. Were original research (case–control, cohort, cross-sectional) or relevant reviews providing mechanistic insights.

Studies were excluded if they:

1. Focused on cancers other than breast cancer without relevant platelet data;
2. Were conference abstracts, editorials, or letters without full data;
3. Lacked sufficient methodological detail or clinical relevance.

## Data extraction and synthesis

Data from eligible studies were extracted using a standardized template, capturing: study design, sample size, patient demographics, platelet indices assessed, outcomes measured (diagnostic accuracy, prognosis, survival), and key findings. Both quantitative and qualitative findings were synthesized narratively, emphasizing consistent patterns, mechanistic insights, and clinical relevance.

## Ethical considerations

As this review was based on previously published literature, ethical approval was not required.

## Limitations of the review method

Being a narrative review, this study did not perform formal meta-analysis or quantitative synthesis. While the search strategy aimed to be comprehensive, selection bias and heterogeneity in study design, sample processing, and platelet measurement may influence interpretations. Nonetheless, this approach allowed integration of mechanistic and clinical perspectives across diverse studies to provide a holistic understanding of platelet indices in breast cancer.

### Platelets beyond hemostasis: biological roles in breast cancer

Traditionally viewed as cellular fragments tasked solely with maintaining vascular integrity and preventing hemorrhage, platelets have emerged as active participants in cancer biology. In breast cancer, their role extends well beyond clot formation, encompassing a wide range of functions that facilitate tumor development, progression, and metastasis. These expanded roles reflect a paradigm shift in our understanding of platelets – not merely as passive responders to vascular injury but as dynamic mediators of tumor–host interactions^[19,20]^. One of the most striking contributions of platelets in breast cancer is their ability to “shield circulating tumor cells (CTCs)” from immune detection. As cancer cells intravasate into the bloodstream, they become vulnerable to shear forces and immune surveillance. Platelets rapidly adhere to these cells, forming protective cloaks that obscure tumor antigens from cytotoxic immune cells such as natural killer (NK) cells. This platelet-mediated immune evasion is a key step in enabling successful hematogenous spread of breast cancer cells to distant organs^[21,22]^. Platelets also actively “promote epithelial-to-mesenchymal transition (EMT),” a process crucial for tumor invasion and metastasis. Through the release of growth factors such as transforming growth factor-beta (TGF-β), platelet-derived growth factor (PDGF), and vascular endothelial growth factor (VEGF), they modulate the tumor microenvironment to favor a more invasive and migratory phenotype. TGF-β, in particular, has been implicated in downregulating epithelial markers and upregulating mesenchymal features, thereby enhancing the metastatic capability of breast cancer cells^[14,23]^.

In addition to promoting migration, platelets contribute significantly to “angiogenesis,” the formation of new blood vessels that supply growing tumors with oxygen and nutrients. Through the storage and release of pro-angiogenic molecules – including VEGF, angiopoietins, and basic fibroblast growth factor (bFGF) – platelets help construct a vascular network that supports tumor expansion and facilitates dissemination. Interestingly, platelets can also transport these factors to premetastatic niches, conditioning distant sites for tumor cell colonization even before cancer cells arrive^[10,24]^.

Furthermore, platelets engage in “bidirectional communication with breast cancer cells.” Tumors can “educate” platelets by altering their RNA and protein profiles, transforming them into more pro-tumoral entities. These so-called tumor-educated platelets (TEPs) exhibit changes in secretion patterns and surface markers, which may further enhance tumor progression and serve as potential biomarkers for disease monitoring^[25–27]^. Beyond these tumor-promoting roles, platelets also influence the “immune landscape” of breast cancer. Their release of chemokines and cytokines can modulate leukocyte recruitment, dendritic cell function, and the polarization of macrophages toward tumor-promoting phenotypes. This immunomodulatory capacity contributes to the immunosuppressive microenvironment that many aggressive breast tumors exploit to avoid host defenses^[28–30]^.

### Thrombopoiesis and platelet indices: clinical parameters with oncologic implications in breast cancer

Thrombopoiesis, the physiological process of platelet production from megakaryocytes in the bone marrow, is a finely regulated cascade influenced by hematopoietic growth factors, systemic inflammation, and marrow microenvironment cues. In the context of breast cancer, this process becomes dysregulated, often hijacked by tumor-derived signals to promote a hypercoagulable, pro-inflammatory, and pro-metastatic state. This altered thrombopoietic landscape is frequently reflected in measurable changes in platelet indices – routinely obtained hematological parameters that can offer insights into disease activity and patient prognosis^[31]^. Among the most studied indices, “mean platelet volume” serves as a surrogate marker of platelet size and reactivity. Larger platelets are metabolically more active and more likely to participate in thrombosis and inflammation. In breast cancer, elevated MPV has been associated with increased tumor aggressiveness, potentially reflecting an upsurge in the production of young, hyperactive platelets driven by interleukin-6 (IL-6) and thrombopoietin. This heightened platelet turnover is not incidental; it is part of a broader systemic response to malignancy that supports tumor progression and immune evasion^[32]^. “Platelet distribution width,” a measure of the variability in platelet size, complements MPV in capturing the heterogeneity of platelet populations in circulation. Elevated PDW in breast cancer patients may indicate asynchronous thrombopoiesis or a reactive marrow responding to tumor-mediated inflammation. This index may also correlate with platelet activation status, as active platelets often undergo shape changes that increase size variability. Together, MPV and PDW form a window into the dynamic alterations in platelet biology during cancer evolution^[33]^.

“Plateletcrit (PCT),” which quantifies the total platelet mass in circulation, has garnered growing interest in oncology. While often underutilized, PCT reflects both platelet count and volume, making it a more integrative measure of thrombopoietic output. In breast cancer, high PCT levels may signal enhanced marrow activity or systemic inflammation, and in some studies, have been linked to more advanced disease and lymph node involvement. Given its relative stability compared to MPV and PDW, PCT may serve as a more consistent marker for long-term disease burden^[34]^. Perhaps the most accessible and widely recognized index, PLT, is frequently elevated in malignancy – a phenomenon termed paraneoplastic thrombocytosis. Breast tumors can stimulate platelet production via cytokine release, particularly IL-6, which acts on hepatic thrombopoietin synthesis and bone marrow stimulation. High platelet counts have been repeatedly associated with poor prognostic features, including larger tumor size, higher grade, and reduced overall survival. These associations underscore the utility of PLT as a broad marker of tumor–host interaction^[35]^. Notably, these indices do not act in isolation. Their real clinical value may lie in their combination or integration with other systemic inflammatory markers, such as the neutrophil-to-lymphocyte ratio (NLR) or platelet-to-lymphocyte ratio (PLR). Such composite markers can enhance prognostic precision, especially in early-stage or triple-negative breast cancers, where traditional biomarkers are limited^[36]^. The appeal of platelet indices lies not only in their biological relevance but also in their “accessibility.” Derived from standard complete blood counts, these parameters require no additional cost or specialized testing, making them especially valuable in low-resource or high-burden settings. However, their interpretation must be contextualized within a broader clinical and hematologic picture, as numerous nonmalignant factors can influence platelet metrics^[37]^.

## Diagnostic and prognostic applications of platelet indices in breast cancer

The use of platelet indices as diagnostic and prognostic biomarkers in breast cancer has attracted growing attention in recent years. These parameters, routinely obtained from complete blood counts (CBCs), provide a cost-effective and minimally invasive means to capture the dynamic interplay between systemic inflammation, thrombopoiesis, and tumor progression. Increasingly, evidence from case–control and cohort studies underscores the clinical significance of PLT, MPV, PDW, and PCT in predicting disease aggressiveness, treatment response, and patient survival outcomes^[38–40]^.

## Platelet count

Thrombocytosis is one of the most consistently reported hematologic abnormalities in breast cancer. Elevated platelet counts reflect tumor-driven cytokine release, particularly IL-6, which stimulates thrombopoiesis. A large prospective cohort by Stone *et al*. demonstrated that patients with platelet counts >350 × 10^9^/L at diagnosis had significantly shorter overall survival (OS) and disease-free survival (DFS). Similarly, Lu *et al*. confirmed that thrombocytosis was strongly associated with advanced stage and aggressive subtypes such as HER2-positive and triple-negative breast cancer. These findings support PLT as an independent prognostic factor and a marker of systemic inflammatory response in breast cancer^[41–43]^.

## Mean platelet volume

MPV, a measure of platelet size and activation, has shown variable but clinically relevant prognostic associations. In a case–control study by Detopoulou *et al*., lower MPV values were observed in patients with advanced disease, suggesting preferential consumption of larger, more reactive platelets in the tumor microenvironment. Conversely, a prospective cohort study by Orellana *et al.* reported higher MPV levels in patients with poor survival outcomes, reflecting increased platelet activation and prothrombotic potential. These conflicting results likely reflect differences in sample processing, disease stage, and methodological design, but collectively indicate that MPV provides insight into platelet activation status and tumor biology^[44–46]^.

## Platelet distribution width

PDW represents heterogeneity in platelet size, which increases during platelet activation and turnover. Several studies have linked elevated PDW with poor prognosis in breast cancer. In a retrospective cohort, Liu *et al.* reported that higher PDW was associated with distant metastasis and reduced DFS. Similarly, Takeuchi *et al*. found that patients with PDW in the highest quartile had nearly twice the risk of recurrence compared with those in the lowest quartile. Therefore, PDW appears to be a strong marker of platelet heterogeneity in response to tumor burden and systemic inflammation^[47,48]^.

## Plateletcrit

Although less frequently studied, PCT – a measure of total platelet mass – has also shown potential clinical value. A case–control study by Hong *et al.* demonstrated that higher PCT levels were associated with larger tumor size, higher stage, and hormone receptor negativity. More recently, Yu *et al*. observed that reductions in PCT during chemotherapy correlated with improved treatment response, suggesting its utility as a dynamic biomarker for monitoring therapy efficacy^[49]^.

## Integrative prognostic significance

The combined assessment of multiple platelet indices appears to enhance predictive accuracy. Yu *et al.* developed a prognostic model integrating PLT, MPV, and PDW, which significantly improved survival prediction compared to individual indices alone. Such integrative approaches highlight the potential of platelet indices to complement established prognostic models, offering a simple and scalable addition to current risk stratification strategies^[49,50]^.

## Mechanistic insights

The interplay between thrombopoiesis and tumorigenesis is underpinned by a complex network of molecular and cellular interactions that modulate both platelet production and function in the context of breast cancer. Mechanistic understanding of these processes is essential to appreciating how platelet indices mirror tumor biology and inform clinical outcomes. One of the central mechanisms linking breast cancer to altered platelet dynamics is the tumor-induced systemic inflammatory response. Breast tumors secrete a variety of cytokines and growth factors – including interleukin-6 (IL-6), interleukin-1β (IL-1β), granulocyte colony-stimulating factor (G-CSF), and vascular endothelial growth factor (VEGF) – which stimulate hepatic production of thrombopoietin (TPO). TPO acts on bone marrow progenitor cells to promote megakaryocyte maturation and platelet release, resulting in elevated platelet counts and increased circulating levels of large, immature platelets. This enhanced thrombopoiesis is reflected in raised PLT, MPV, and possibly PCT values observed in many breast cancer patients^[51]^. Beyond increased production, tumor-induced platelet activation plays a critical role in supporting malignant behavior. Tumor cells interact directly with platelets through surface molecules, such as P-selectin and integrins, leading to platelet activation and degranulation. Activated platelets release a host of bioactive mediators – including platelet-derived growth factor (PDGF), transforming growth factor-beta (TGF-β), and serotonin – that promote tumor cell proliferation, angiogenesis, and epithelial-to-mesenchymal transition (EMT). These factors not only support primary tumor growth but also prime the metastatic cascade by enhancing tumor cell motility and survival in circulation^[52]^.

**Figure 1.**
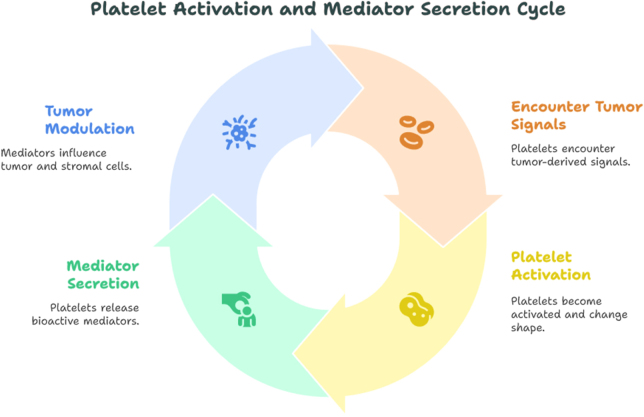
Mechanistic insights: platelet activation and mediator secretion in breast cancer.

Circulating tumor cells (CTCs), once intravasated into the bloodstream, are rapidly cloaked by activated platelets. This platelet “shield” protects CTCs from shear stress and immune surveillance, particularly by natural killer (NK) cells, facilitating their survival during transit to distant sites. Moreover, platelet-derived TGF-β and microparticles contribute to the remodeling of distant tissue niches, creating a hospitable microenvironment for metastatic colonization. These platelet-mediated events are intimately tied to platelet size variability and activation state, correlating with elevated PDW and fluctuating MPV in patients with advanced or metastatic breast cancer^[53]^. Another key mechanism involves the reciprocal interaction between tumor-derived extracellular vesicles (EVs) and platelets. Tumor EVs can induce platelet activation and aggregation, while platelet-derived microparticles (PMPs) can transfer oncogenic RNA and proteins back to tumor or stromal cells, thus perpetuating a pro-tumorigenic feedback loop. This bidirectional communication further underscores the dynamic role of platelets as both responders to and facilitators of cancer progression^[19,54]^. Furthermore, recent studies suggest that platelets may carry tumor-specific transcripts and proteins, functioning as “circulating mirrors” of tumor activity – a concept encapsulated in the emerging field of “tumor-educated platelets” (TEPs). Although not yet reflected in routine clinical indices, these findings reinforce the biological plausibility of using platelet-derived parameters as surrogates for disease activity (Fig. 1)^[55]^.

## Platelets as carriers of tumor RNA and mutated proteins

Beyond their traditional role in hemostasis, platelets have emerged as dynamic carriers of tumor-derived molecular information, including RNA transcripts and mutated proteins. This property positions them as both facilitators of tumor progression and valuable sources of biomarkers in breast cancer. Tumor cells release various bioactive molecules and extracellular vesicles, which are taken up by circulating platelets. Consequently, platelets acquire tumor-specific RNA, such as messenger RNA (mRNA) and microRNA (miRNA), as well as mutated proteins that mirror the genetic landscape of the malignancy^[56]^. Mechanistically, platelets interact with tumor cells through direct contact, soluble mediators, and vesicle-mediated transfer. Once internalized, these tumor-derived components can alter platelet behavior, enhancing their ability to support angiogenesis, immune evasion, and metastatic dissemination. For instance, platelet-mediated delivery of oncogenic RNA may facilitate communication between tumor cells and distant microenvironments, preparing premetastatic niches and promoting metastatic competence^[57]^.

Clinically, the ability of platelets to carry tumor-specific RNA and mutated proteins has significant diagnostic and prognostic implications. Several studies have demonstrated that platelet RNA profiles can discriminate breast cancer patients from healthy controls with high sensitivity and specificity. These profiles often reflect tumor subtype, stage, and even treatment response, making them attractive candidates for minimally invasive liquid biopsy approaches. In addition, the detection of mutated proteins within platelets offers complementary information, potentially revealing actionable oncogenic alterations that guide targeted therapy^[58]^. From a prognostic perspective, the quantity and type of tumor-derived material within platelets correlate with disease aggressiveness. Elevated levels of pro-metastatic RNA transcripts or mutated proteins in platelets have been associated with increased risk of recurrence and poorer survival outcomes. Thus, platelet cargo not only mirrors tumor biology but may also serve as an early indicator of disease progression or therapeutic resistance^[59]^. Therapeutically, understanding the mechanisms of RNA and protein transfer between tumor cells and platelets opens avenues for intervention. Strategies aimed at disrupting this molecular exchange, or modulating the functional consequences of tumor cargo within platelets, could potentially reduce metastatic spread and enhance treatment efficacy. Moreover, integrating platelet-derived biomarkers into clinical practice offers a noninvasive, real-time method to monitor tumor evolution and personalize breast cancer management^[60]^.

## Tumor-educated platelets

The concept of tumor-educated platelets (TEPs) has gained increasing attention as a novel frontier in cancer biology, with significant implications for breast cancer diagnosis, prognosis, and therapy. TEPs refer to circulating platelets that undergo functional and molecular reprogramming under the influence of tumor-derived signals, including cytokines, growth factors, extracellular vesicles, and microRNAs. Through this bidirectional interaction, breast cancer cells exploit platelets to facilitate tumor progression, angiogenesis, and metastatic spread, while platelets in turn acquire distinct transcriptomic and proteomic signatures reflective of the tumor microenvironment^[60]^. Mechanistically, breast cancer cells release bioactive molecules such as VEGF, PDGF, and TGF-β, which alter the platelet activity, granule content, and RNA profiles. Platelets then act as “messengers” that not only shield circulating tumor cells (CTCs) from immune clearance but also enhance their adhesion to vascular endothelium, thereby promoting extravasation and metastasis. Importantly, the molecular reprogramming of platelets is not random but highly specific, allowing them to serve as sensitive biosensors of tumor presence and dynamics^[19,61]^.

From a diagnostic standpoint, TEPs hold promise as a liquid biopsy tool. Multiple studies have demonstrated that the RNA profiles of TEPs can discriminate cancer patients from healthy individuals with high accuracy, including those with early-stage breast cancer. This opens opportunities for minimally invasive screening strategies, particularly in resource-limited settings where advanced imaging or tissue biopsies may not be readily available. Furthermore, longitudinal monitoring of TEPs has been shown to reflect tumor burden and therapeutic response, offering an advantage over static biomarkers such as CA15-3^[62]^. Prognostically, the altered gene expression patterns and secretory activity of TEPs correlate with tumor aggressiveness, metastatic potential, and survival outcomes. Patients with higher levels of platelet activation markers or TEP-derived pro-metastatic factors often exhibit reduced progression-free and overall survival. Incorporating TEP analysis into clinical practice could therefore refine risk stratification, enabling the identification of patients who may benefit from intensified treatment or closer follow-up^[63]^. Therapeutically, TEPs present an innovative target for intervention. Strategies aimed at disrupting tumor–platelet crosstalk, modulating TEP-derived signaling pathways, or inhibiting the release of platelet-derived RNA cargo are currently under exploration. Additionally, TEP profiling may help identify novel therapeutic targets within breast cancer biology itself, bridging the gap between diagnostics and therapeutics^[64]^.

## Clinical implications for treatment

The emerging role of platelet indices in breast cancer not only provides prognostic insights but also carries important implications for clinical management and treatment strategies. Since platelet parameters can be measured routinely and at low cost, they represent practical tools that could complement existing diagnostic and therapeutic frameworks^[65]^. From a treatment-monitoring perspective, dynamic changes in platelet indices during systemic therapy may serve as early markers of response. For example, reductions in PCT and PLT following chemotherapy have been correlated with favorable treatment outcomes, whereas persistently elevated values may indicate resistant disease or higher risk of relapse. This suggests that routine monitoring of platelet indices could help clinicians tailor treatment intensity and anticipate therapeutic failure earlier than conventional imaging or tumor markers^[66]^. Furthermore, high platelet counts and increased platelet distribution width (PDW) at diagnosis may identify patients at greater risk for metastasis and poor survival. These patients could benefit from closer surveillance, more aggressive systemic therapy, or adjunctive interventions to counteract platelet-mediated tumor progression. In this context, integrating platelet indices into established prognostic scoring systems, such as the Nottingham Prognostic Index, could refine risk stratification and improve individualized treatment planning^[67]^.

**Figure 2.**
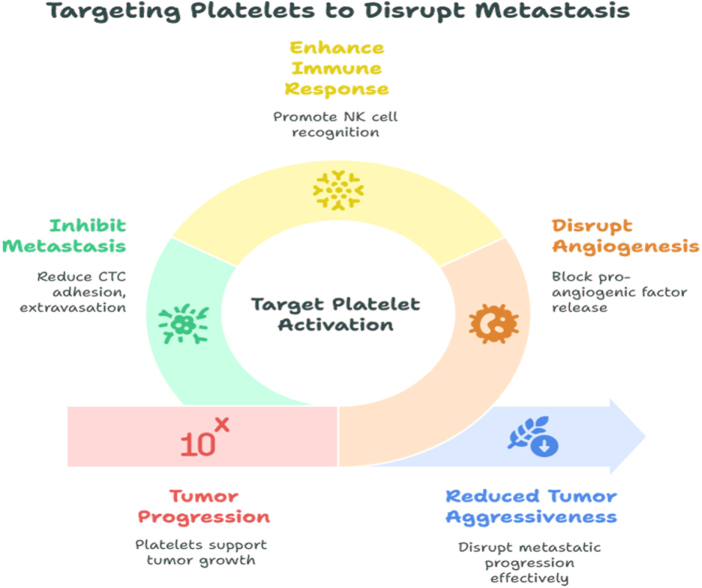
Platelet dynamics in tumor angiogenesis, immune evasion, and metastasis.

The interplay between platelets and the tumor microenvironment also highlights opportunities for therapeutic modulation. Antiplatelet therapies such as aspirin or P2Y_12_ inhibitors, when carefully evaluated, could complement standard cancer treatments by limiting platelet-driven angiogenesis, immune evasion, and metastasis. Although further clinical trials are required to confirm efficacy and safety, these approaches are particularly attractive in patients with co-existing cardiovascular comorbidities, where dual benefits may be achieved^[68]^. Importantly, platelet indices may also have implications for supportive care in breast cancer. Given the increased risk of VTE in this population, monitoring platelet dynamics can help identify patients at heightened risk and guide thromboprophylaxis decisions. This is especially relevant in settings where advanced coagulation testing is not available, making platelet indices a cost-effective surrogate marker of thrombotic risk (Fig. 2) ^[69,70]^.

## Platelets as targets in breast cancer therapy

The recognition that platelets are not passive bystanders but active participants in breast cancer progression has opened new avenues for therapeutic intervention. Beyond their role in hemostasis, platelets facilitate tumor cell survival in circulation, promote angiogenesis, and aid metastatic colonization. Targeting these platelet–tumor interactions therefore represents a promising adjunctive strategy in breast cancer therapy^[57]^. Several approaches have been investigated to modulate platelet function in the oncological setting. The use of antiplatelet agents, particularly aspirin, has been widely studied due to its ability to irreversibly inhibit platelet cyclooxygenase-1 (COX-1) and reduce thromboxane A_2_ synthesis. Epidemiological studies and meta-analyses suggest that regular aspirin use may be associated with a reduced incidence of breast cancer and lower risk of distant metastasis, although clinical outcomes vary depending on dose, timing, and tumor subtype^[71,72]^.

Another emerging therapeutic concept is the use of P2Y^12^ receptor antagonists, such as clopidogrel and ticagrelor, which block ADP-mediated platelet activation. Preclinical models indicate that inhibition of P2Y^12^ signaling reduces platelet–tumor aggregation and impairs metastatic spread. Translational studies are currently exploring whether such strategies could complement standard breast cancer therapies^[73,74]^. Beyond conventional antiplatelet drugs, researchers are investigating platelet-derived growth factor (PDGF) and VEGF pathways as therapeutic targets. These platelet-secreted factors are central to tumor angiogenesis and stromal remodeling. The integration of anti-angiogenic agents, such as bevacizumab (anti-VEGF), into treatment regimens has demonstrated the clinical relevance of platelet-mediated angiogenesis, although resistance mechanisms remain a challenge^[75]^.

Another novel approach involves TEP), which carry tumor-derived RNA and proteins. TEPs could be exploited both diagnostically – as a form of liquid biopsy – and therapeutically, by disrupting tumor–platelet cross-talk. For instance, strategies that interfere with the transfer of tumor-derived microparticles to platelets are being evaluated as potential means to attenuate metastatic competence^[60]^. It is also noteworthy that platelets influence the immune landscape of breast cancer. By shielding tumor cells from natural killer (NK) cell-mediated lysis, platelets contribute to immune evasion. Therefore, combining platelet-targeted interventions with immunotherapy may enhance antitumor immune responses. Early-phase studies suggest that platelet inhibition could augment the efficacy of immune checkpoint inhibitors in preclinical breast cancer models^[76,77]^.

## Emerging evidence on modulating thrombopoiesis and platelet activity in breast cancer

Recent clinical and preclinical studies have generated growing interest in how modulation of thrombopoiesis and platelet activation shapes the tumor microenvironment, influences tumor–platelet interactions, and ultimately affects metastatic progression in breast cancer. Tumor-associated inflammation stimulates megakaryopoiesis through cytokines such as IL-6 and thrombopoietin, resulting in increased production of larger, metabolically active platelets with enhanced proangiogenic and immunomodulatory potential. Experimental models show that restricting thrombopoiesis or reducing platelet reactivity can alter the stromal composition of the breast tumor microenvironment, reducing levels of VEGF, TGF-β, and PDGF that normally facilitate angiogenesis, epithelial–mesenchymal transition (EMT), and extracellular matrix remodeling. These mechanistic findings underscore thrombopoiesis as a potentially modifiable axis in breast cancer biology^[73,74]^.

Preclinical studies using genetic and pharmacologic approaches to suppress megakaryocyte activity or limit platelet activation have demonstrated reductions in circulating tumor cell survival, impaired metastatic seeding in the lungs, and decreased tumor vascular density. For example, mouse models lacking functional thrombopoietin signaling exhibit attenuated metastatic potential, suggesting a direct role of thrombopoiesis in sustaining pro-metastatic platelet–tumor crosstalk. Similarly, inhibition of platelet-derived microparticle release has been shown to limit the transfer of pro-tumorigenic cargo, including microRNAs and growth factors, thereby disrupting tumor adaptation to hostile microenvironmental conditions^[75]^.

Parallel to these mechanistic insights, a growing body of clinical research has explored antiplatelet therapies as a means of modulating tumor progression. Aspirin remains the most widely studied agent, with observational data suggesting that long-term use may reduce breast cancer–specific mortality, especially in hormone receptor–positive disease. Aspirin’s inhibition of COX-1 and subsequent reduction of thromboxane A2 limits platelet activation and aggregation, potentially impairing platelet-mediated shielding of circulating tumor cells and diminishing metastatic spread. Early-phase trials evaluating aspirin as an adjuvant agent have reported favorable effects on biomarkers of platelet activation, although definitive outcomes on tumor progression remain under investigation^[76]^.

P2Y12 inhibitors, including clopidogrel and ticagrelor, have also gained attention for their ability to block ADP-mediated platelet activation. Emerging preclinical data indicate that P2Y12 blockade reduces tumor cell-induced platelet aggregation and disrupts the formation of platelet–tumor microthrombi critical for metastatic niche formation. Preliminary translational findings suggest potential synergy between P2Y12 inhibitors and standard chemotherapeutic agents, though large-scale clinical trials in breast cancer are still lacking^[77]^. In addition to classic antiplatelet agents, targeted therapies aimed at platelet-derived growth pathways have begun to show promise. Experimental inhibition of PDGF, VEGF, and TGF-β signaling has rev ealed downstream effects on tumor proliferation, angiogenesis, and stromal remodeling. Some early-phase trials evaluating anti-VEGF or anti-PDGF agents in advanced breast cancer indicate modest improvements in progression-free survival, particularly when combined with cytotoxic or endocrine therapy. While not designed specifically to modulate thrombopoiesis, these agents indirectly alter platelet–tumor signaling axis components, reinforcing the concept that platelet-derived factors are central to breast cancer progression^[76,77]^.

## Conclusion

The intersection of thrombopoiesis and tumorigenesis presents a promising frontier in breast cancer research and clinical practice. Platelets, long recognized for their role in hemostasis, have emerged as active participants in cancer progression, influencing angiogenesis, immune modulation, and metastatic spread. Alterations in platelet indices – including PLT, MPV, PDW, and PCT – reflect these underlying biological processes and offer a window into the systemic response to malignancy. Current evidence underscores the potential of these routinely available hematologic markers to serve as adjunctive tools in the diagnosis, prognostication, and therapeutic monitoring of breast cancer. Their accessibility, low cost, and non-invasive nature make them especially attractive in resource-limited settings and for repeated assessments over the course of disease management. While elevated PLT and PDW have been most consistently associated with advanced disease and poor outcomes, the prognostic value of MPV and PCT remains an area of active investigation.
